# *Mogibacterium* bacteraemia in an elderly woman with severe periodontal disease

**DOI:** 10.1016/j.nmni.2025.101585

**Published:** 2025-04-09

**Authors:** Victoria Jordan, Syeda Naqvi

**Affiliations:** a Department of Microbiology, NSW Health Pathology, John Hunter Hospital, New Lambton Heights, NSW, 2305, Australia; b School of Medicine and Public Health, University of Newcastle, Callaghan, NSW, Australia

**Keywords:** *Mogibacterium*, *Mogibacterium timidum*, Periodontal disease, Anaerobic bacteraemia

Dear editor,

*Mogibacterium* spp. are uncommonly encountered, strictly anaerobic gram-positive rods of unclear pathogenic potential. Here, we present the case of an elderly lady with severe periodontal disease leading to *Mogibacterium* bacteraemia, the first described case of invasive disease with this organism.

A woman in her 70s presented after a syncopal episode on a background of left lower toothache for 1–2 weeks. She had poor dentition with 5–6 teeth remaining, and a background of dementia, osteoarthritis, gastro-oesophageal reflux disease, and hypertension. On examination, she was afebrile and hemodynamically stable but was noted to have a delirium and mild swelling over the left lower jaw. Investigations revealed elevated inflammatory markers with a neutrophil count of 17.0 x 10^9^/L (reference range 2.0–8.0 x 10^9^/L) and C-reactive protein (CRP) of 178 mg/L (reference range <5 mg/L). Two sets of blood cultures were collected, and she was commenced on oral amoxicillin-clavulanate 875mg/125mg twice daily. 48 hours later, though afebrile and mental status back to baseline, she had ongoing jaw pain and an uptrending CRP to 199 mg/L; she was discharged from hospital on her request, with 5 further days of amoxicillin-clavulanate.

A blood culture from admission subsequently flagged positive at 72 hours with growth from the anaerobic bottle only, appearing as straight, medium-sized gram-positive rods (Fig. 1). Growth from subculture occurred only anaerobically, appearing at 48 hours as pinpoint, translucent-grey colonies. The organism was identified as *Mogibacterium timidum* using Matrix Associated Laser Desorption Ionisation Time of Flight (MALDI-TOF; Bruker Biotyper, Bruker, MA, USA) with a maximum score of 1.97 SV. The organisms were non-motile and catalase negative. On subculture, growth was optimal on anaerobe agar compared with horse blood and chocolate agar, though over time colonies did not significantly increase in size, remaining tiny by day 7. The organism was unfortunately non-viable for antimicrobial susceptibility testing.

**Fig. 1:**
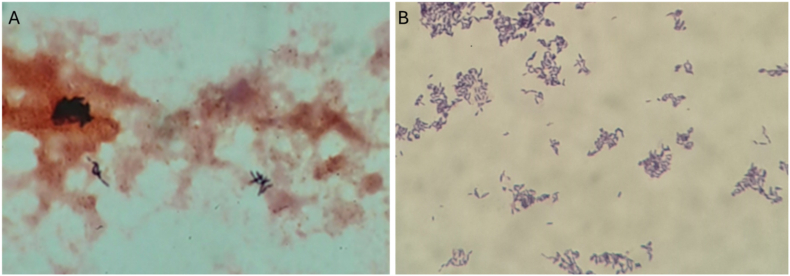
Gram stains from a) blood culture bottle and b) a colony of *Mogibacterium timidum.*

The patient was readmitted 9 days following the positive blood culture with ongoing jaw pain and swelling. In the interim, she had undergone dental review and required a filling and clean. Contrast-enhanced computed tomography (CT) of the jaw showed periodontal disease adjacent to the left lower canine with mild soft tissue thickening however no abscess or osteomyelitis. She was treated with 10 days total (intravenous and oral) amoxicillin-clavulanate. Follow up bloods a week later showed negative blood cultures and normalised inflammatory markers, with improved though ongoing dental pain for which she requires ongoing dental input.

The *Mogibacterium* genus was described in the year 2000 as non-spore forming, non-motile, strictly anaerobic gram-positive rods [1]. *Mogibacterium timidum* was reclassified into the genus from the already described *Eubacterium timidum* after phylogenetic re-analysis [1]. There are several species including *M. timidum*, *M. pumilum*, *M*. *vescum* [1], *M. kristiansenii*, *M. neglectum*, and *M. diversum.* The genus has been found as a component of human and porcine gastrointestinal microbiota, and one study assessing microbiome risk factors associated with the colorectal cancer found *Mogibacterium* spp*.* prevalence to be relatively increased in the intestinal microbiota of colorectal cancer patients compared with healthy controls [2]. Otherwise, the only descriptions of *Mogibacterium* spp. in human disease have been in the context of dental disease, including plaque, abscess, periodontal disease, and infected root canals, and osteonecrosis of the jaw [3]. Whether it is directly pathogenic in these cases or a marker of dysbiosis or underlying disease is unclear [3].

A 2022 study evaluating bacteria associated with descending necrotising mediastinitis versus uncomplicated maxillofacial infection by sequencing the pus samples of these patients found *Mogibacterium* spp. in the top 10 bacteria detected in the patients who developed necrotising mediastinitis, but not in the top 10 of the patients with uncomplicated infection [4]. However, the literature is scarce beyond this on detection of *Mogibacterium* spp., cultured or otherwise, in association with any other site of infection. There is therefore no guidance on empirical antibiotic regimens for invasive disease, and certainly no clinical breakpoints or susceptibility testing methodology are described by either the European Committee on Antimicrobial Susceptibility Testing (EUCAST) nor the Clinical and Laboratory Standards Institute (CLSI) [5,6].

Our patient had signs of treatment success in clearance of bacteraemia, reduced swelling and downtrending inflammatory markers after amoxicillin-clavulanate and minor dental intervention, however it is possible that the bacteraemia represented a transient translocation, and antimicrobial susceptibility cannot be inferred. Furthermore, the ongoing presence and relative abundance of *Mogibacterium* in her oral flora is unknown. In conclusion, *Mogibacterium* spp. are uncommonly encountered organisms but likely prevalent in oral flora, particularly in patients with periodontal disease. Invasive disease is rare but possible particularly in severe disease. More evidence is required to inform in vitro susceptibility testing methodology and optimal treatment.

## CRediT authorship contribution statement

**Victoria Jordan:** Writing – original draft, Writing – review & editing. **Syeda Naqvi:** Conceptualization, Supervision, Writing – review & editing.

## Ethics

Consent was obtained from the patient’s legal Guardian.

## Funding

This work received no specific grant from any funding agency.

## Declaration of competing interest

The authors declare that they have no known competing financial interests or personal relationships that could have appeared to influence the work reported in this paper.
